# Killer Whale Nuclear Genome and mtDNA Reveal Widespread Population Bottleneck during the Last Glacial Maximum

**DOI:** 10.1093/molbev/msu058

**Published:** 2014-02-04

**Authors:** Andre E. Moura, Charlene Janse van Rensburg, Malgorzata Pilot, Arman Tehrani, Peter B. Best, Meredith Thornton, Stephanie Plön, P.J. Nico de Bruyn, Kim C. Worley, Richard A. Gibbs, Marilyn E. Dahlheim, Alan Rus Hoelzel

**Affiliations:** ^1^School of Biological and Biomedical Sciences, Durham University, Durham, United Kingdom; ^2^Department of Zoology and Entomology, Mammal Research Institute, University of Pretoria, Hatfield, Pretoria, South Africa; ^3^Eurofins MWG Operon, Ebersberg, Germany; ^4^Department of Zoology and Entomology, Mammal Research Institute, University of Pretoria, c/o Iziko South African Museum, Cape Town, South Africa; ^5^South African Institute for Aquatic Biodiversity (SAIAB), c/o PE Museum/Bayworld, Humewood, Port Elizabeth, South Africa; ^6^Department of Molecular and Human Genetics, Baylor College of Medicine, Houston, TX; ^7^National Marine Mammal Laboratory, National Marine Fisheries Service, Seattle, WA

**Keywords:** genomics, demographics, Cetacea, population bottleneck

## Abstract

Ecosystem function and resilience is determined by the interactions and independent contributions of individual species. Apex predators play a disproportionately determinant role through their influence and dependence on the dynamics of prey species. Their demographic fluctuations are thus likely to reflect changes in their respective ecological communities and habitat. Here, we investigate the historical population dynamics of the killer whale based on draft nuclear genome data for the Northern Hemisphere and mtDNA data worldwide. We infer a relatively stable population size throughout most of the Pleistocene, followed by an order of magnitude decline and bottleneck during the Weichselian glacial period. Global mtDNA data indicate that while most populations declined, at least one population retained diversity in a stable, productive ecosystem off southern Africa. We conclude that environmental changes during the last glacial period promoted the decline of a top ocean predator, that these events contributed to the pattern of diversity among extant populations, and that the relatively high diversity of a population currently in productive, stable habitat off South Africa suggests a role for ocean productivity in the widespread decline.

## Introduction

Environmental change through anthropogenic or natural processes can drive population dynamics and patterns of distribution, and apex predators are sentinel species reflecting the impact of change and influencing ecosystem dynamics (Sergio et al. 2005; Myers et al. 2007; Sergio et al. 2008). For example, the depletion of top predatory shark species led to cascading effects that increased the abundance of mesopredators (Myers et al. 2007), and model predictions suggest that major habitat shifts for marine top predators in the Pacific could result from future climate warming, with up to 35% changes in core habitat and a significant displacement of biodiversity (Hazen et al. 2012). Here, we consider the historical population dynamics of a species at the top of ocean trophic chains, the killer whale (*Orcinus orca*), and the implications for past and future ocean ecosystems in the context of environmental change. The killer whale is a worldwide distributed cetacean which feeds on a variety of prey, including fish, pinnipeds, other cetaceans and sharks (Matkin and Leatherwood 1986). Regional populations in matrifocal social groups (called pods) often specialize by prey choice or foraging strategy, defining ecotypes such as the fish-eating and marine-mammal-eating ecotypes in the North Pacific (Hoelzel et al. 2007).

Although various factors are likely to influence the abundance and distribution of a species in space and time (including competition and the abundance and distribution of predators and prey), for cetaceans various studies have proposed an influence of climate change in particular. For example, a correlation between climatic cycles and the appearance of new species or differentiated populations has been described for a range of taxa (Hoelzel, Potter, et al. 1998; Natoli et al. 2005; Hoelzel et al. 2007; Whitehead et al. 2008; Steeman et al. 2009; Amaral et al. 2012). More directly, distributional and abundance changes have been documented based on recent climate changes, such as for the Pacific white-sided dolphin (*Lagenorhynchus obliquidens*) (Salvadeo et al. 2010), or on historical patterns of climate change, as for bowhead whales (*Balaena mysticetus*) in the North Atlantic during the Holocene (Foote et al. 2013). For the killer whale, one study suggested redistribution and the founding of new populations associated with the release of coastal habitat after the last glacial maximum (LGM) (Hoelzel et al. 2007). Also relevant in this context are studies that found low worldwide genetic diversity for killer whales at both mitochondrial and nuclear DNA markers (Hoelzel, Natoli, et al. 2002; Morin et al. 2010), which was variously interpreted as cultural hitchhiking of mtDNA along highly social matrilines (Whitehead 1998) or evidence for an historical population bottleneck (Hoelzel, Natoli, et al. 2002).

There is a long-standing debate about the relative importance of climate change versus human hunting in forcing major events such as the late Pleistocene megafaunal extinctions (Owen-Smith 1987; Barnosky 2004). A detailed review by Barnosky et al. (2004) concluded that in terrestrial systems anthropogenic impact was likely to have been significant. This might account for the observation that such an extreme event as the megafaunal extinction associated with the end of the Pleistocene, when half of known genera of large terrestrial mammals went extinct, was not evidently preceded by major extinction events during earlier glacial cycles (Owen-Smith 1987). However, while oceanic species including cetaceans have been heavily impacted by human activities in recent times, human interference could have only realistically impacted coastal populations during the Pleistocene to Holocene transition (Matkin and Leatherwood 1986).

Coalescent methods that trace past demographic histories have provided important inference about the possible role and relative importance of climate change on natural populations. For example, the Beringian plains bison (*Bison cf. priscus*) was shown to decline prior to the arrival of humans at a time of rapid climate change (Shapiro et al. 2004), and the southern elephant seal (*Mirounga leonina*) rapidly responded to the gain and loss of breeding habitat when Holocene climate led to the retreat and advance of Antarctic ice (de Bruyn et al. 2009). In general, it may be expected that rapid periods of change (such as the terminations leading into interglacials) or extended periods of cold weather during glacial phases of the Pleistocene Milankovitch cycles, would be the most likely events associated with population decline and extinctions. Consistent with this, there are various declines that can be associated either with the LGM or climatic events during the Holocene (see review in de Bruyn et al. [2011]). There are, however, also species-specific effects, where changes in habitat suitability apparently promoted growth rather than decline through climatic transitions (de Bruyn et al. 2009; Hofreiter and Stewart 2009; Foote et al. 2013). Redistributions of fauna during the LGM have been described in both terrestrial (Hewitt 2004) and marine systems (Banguera-Hinestroza et al. 2010; Allcock and Strugnell 2012), typically associated with population decline into glacial refugia and reexpansion during subsequent periods of global warming. The potential for this being a repeating cycle throughout the Pleistocene climate cycles (with the phylogeographic signal lost during the interglacials) was suggested by terrestrial work with ancient DNA from the Eemian interglacial (Hofreiter 2004).

Here, we generate a 2.23-Gb draft killer whale genome sequence at 20× coverage, and apply the pairwise sequentially Markovian coalescent (PSMC) model (Li and Durbin 2011) to two genomes (our sequence from the North Pacific and a database killer whale sequence from the North Atlantic; prepublication access to the North Atlantic sequence provided by coauthors K.C.W. and R.A.G.) to assess demographic history over the Pleistocene timeframe. We also investigate mtDNA genetic diversity for samples collected worldwide (*N* = 616), including a previously unsampled geographic region off the coast of southern Africa, where oceanic conditions are understood to have been relatively stable throughout the Pleistocene. We test two key hypotheses. First, we test the hypothesis that the historical demography of a marine apex predator would track patterns of climate change that in turn may have impacted resource availability. Second, given evidence for a population bottleneck, we test the hypothesis that local, environmentally stable marine regions may have retained refugial populations of the killer whale that retained historical diversity.

## Results

**Fig. 1.**
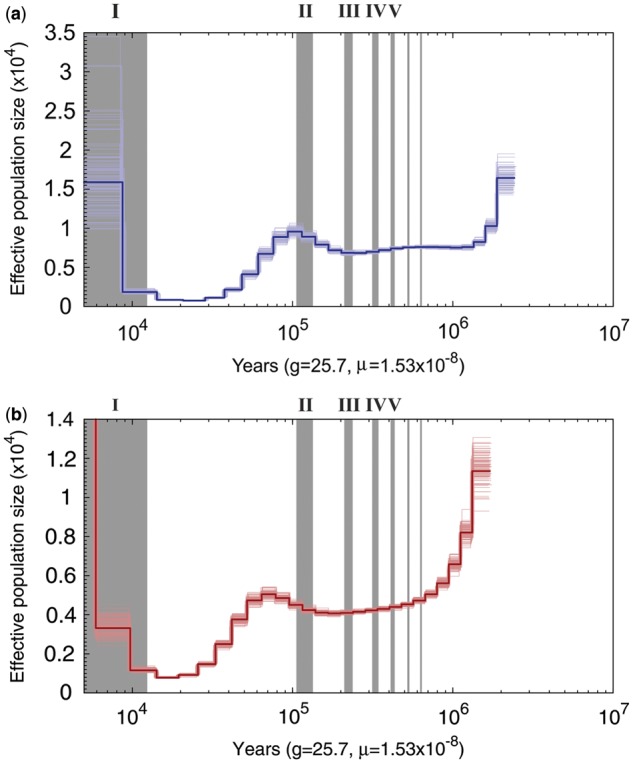
PSMC plot for North Pacific killer whale genome (*a*) and North Atlantic sequence (*b*) from Genbank (SRA058929; note difference of scale—adjusted to make confidence limits clear) showing confidence limits. Gray bars indicate the interglacial periods following Pleistocene terminations (indicated by Roman numerals). Time ranges for interglacial periods displayed (in years before present): I: 0–14,000; II: ∼115,000–130,000; III: ∼220,000–243,000; IV: ∼320,000–337,000; V: ∼400,000–424,000. *g* and *μ* represent the generation time and mutation rate (/nucleotide site/year) assumed, respectively.

Using the PSMC method, we find that killer whale effective population size (Ne) strongly declined during the last glacial period and remained low into the early Holocene (fig. 1). The longer term dynamics reflect a relatively stable population throughout the periodic glacial cycles of the middle-late Pleistocene. A possible population expansion into the Holocene (during a period of global warming) is suggested, but this is more convincingly implied from earlier mtDNA studies (Hoelzel, Natoli, et al. 2002) and our mismatch analysis (discussed later). Our North Pacific sample (SRP035610) and a draft genome from a North Atlantic sample (SRA058929) show essentially the same profile, though the long-term Ne is somewhat lower in the North Atlantic sample (fig. 1). Note that little resolution is possible for the relatively recent and older time periods, such that neither the apparent demographic increase into the Holocene nor the decrease into the Pleistocene is likely to be robust from this analysis (Li and Durbin 2011). Uncertainty over an appropriate genomic mutation rate (Dornburg et al. 2012) reduces temporal precision of the inferred demographic profile; however, our interpretation is supported within a credible range of values (supplementary fig. S1, Supplementary Material online; see Materials and Methods). Changes in connectivity can also affect these estimates (e.g., if newly connected populations reflect the deeper coalescence of the combined populations), but would be unlikely to generate the specific pattern observed (strong population decline) or the very similar profiles for each ocean.

**Fig. 2.**
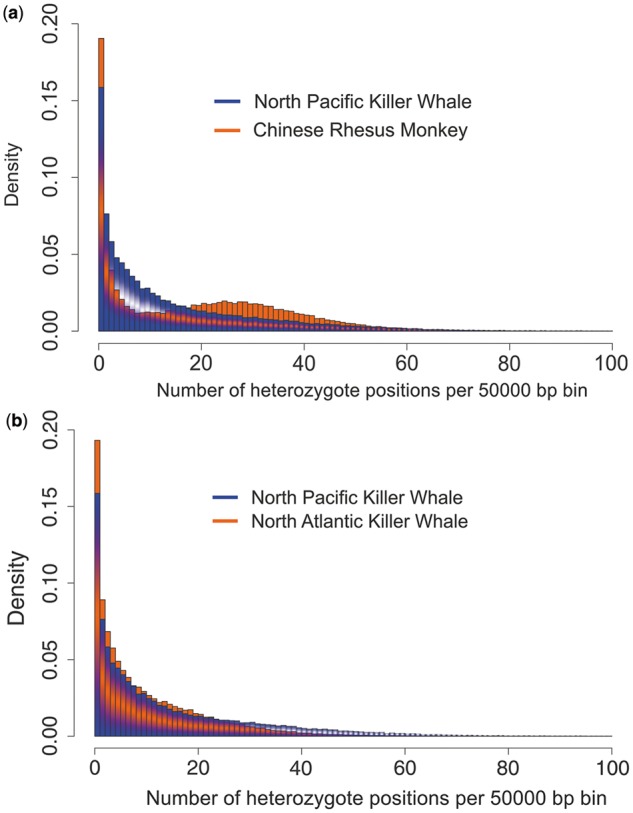
Density distributions for heterozygous sites per 50,000-bp bins for draft genome sequences comparing the killer whale and rhesus monkey (*a*) and comparing the two killer whale genomes (*b*).

Diversity across genomes are compared between the killer whale and Chinese rhesus monkey (*Macaca mulatta*) in figure 2, and including the North Atlantic killer whale (also thought to be from a piscivorous ecotype) in figure 2*b* and table 1. The Chinese rhesus monkey has a relatively large and stable population and shows approximately 3-fold higher genomic diversity than humans (but estimated mutation rates are not higher than for cetaceans; Yuan et al. 2012). Both show a peak of low diversity regions, representing highly conserved DNA (North Pacific killer whale shown in fig. 2*a*, the nearly identical profiles for the two killer whales compared in fig. 2*b*), and only the rhesus monkey has a second peak reflecting more diverse regions (and overall higher diversity, despite possible undersampling of heterozygotes due to the relatively low 6× coverage [Yuan et al. 2012], see Materials and Methods). Each plot reflects the genomic diversity of just one individual. Although diversity levels will vary among individuals in natural populations, the killer whales show very similar median diversity, each lower than the rhesus monkey (table 1). Diversity levels among the genomes of 14 species were compared by Cho et al. (2013) as the rate of heterozygous single nucleotide variants, including rare and endangered species such as the snow leopard. The two killer whale genomes had similar values (0.00036 for the North Atlantic and 0.00043 for the North Pacific) to that seen for the white lion and Amur tiger (0.0005), Tasmanian devil (0.0003), and snow leopard (0.00025). From figure 1, the killer whale population low point is suggested to be Ne= ∼900, lasting for ∼1,600 killer whale generations, which would reduce variation by ∼60% based on drift alone in a single panmictic population (whereby heterozygosity in generation *t* (*H_t_*) is a function of the initial heterozygosity (*H_0_*), the number of generations and the effective population size; *H_t_* = *H_0_*(1 − (1/2Ne)*^t^*)). Natural subdivision and founder events (discussed later) may have reduced diversity further.

**Table 1.** Parameters from Comparison Illustrated in Figure 2 (heterozygote positions per 50,000-bp bins).

	Mean	Median	Mode	Maximum
NP killer whale	15.56	9	1	663
NA killer whale	10.98	6	1	484
Monkey	19.08	17	1	600

**Fig. 3.**
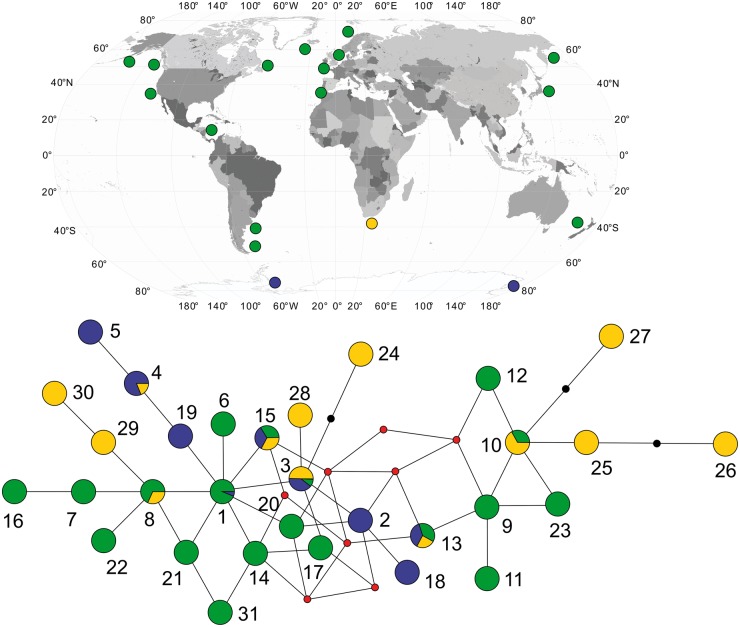
Median joining network based on mtDNA control region fragment. Illustration shows the relationship among haplotypes, but not haplotype frequencies (see supplementary table S1 [Supplementary Material online] which lists haplotype names and accession numbers by number illustrated in the figure), with yellow indicating South African samples, blue Antarctic, and green the rest of the world. The red dots indicate hypothetical haplotypes not found in the data set, while the black dots indicate that there are two steps in those branches. The figure shows the location of global sample sites (see supplementary tables S1 and S2 [Supplementary Material online] for more detail).

Figure 3 provides a mtDNA median joining network for the 31 haplotypes representing 616 samples from around the world based on the 300-bp control region fragment (see Materials and Methods), including 13 haplotypes found among 37 whales from South African waters (supplementary table S1, Supplementary Material online). Networks for regional populations showing the relative proportion of haplotypes are provided in supplementary figure S2, Supplementary Material online. When the South African samples are compared against the database sequences, there are 20 segregating sites and 14 for the South African samples alone. All changes were transition mutations. The high diversity (gene diversity, *π* = 0.0147 ± SD 0.0008; haplotypic diversity, *h* = 0.889 ± 0.030), incidence of private haplotypes (7 of the 13 haplotypes found for South Africa), and distribution of South African haplotypes throughout the network (including shared central nodes) suggests a refugial population that retained ancestral diversity lost in other populations. The South African population also shows greater evidence of stability over time based on network shape (showing multiple haplotypes with relatively even proportions; supplementary fig. S2, Supplementary Material online). Although some regions remain undersampled (especially South America and New Zealand where further refugia are possible), most regions share the same or similar haplotypes (e.g., full control region sequences match between the eastern North Atlantic and New Zealand [Hoelzel, Natoli, et al. 2002]), unlike South Africa where most haplotypes are private or rare (fig. 3; supplementary table S1, Supplementary Material online).

Diversity elsewhere ranged from a low among the North Pacific “resident” ecotype (*π* = 0.0013 ± 0.0001; *h* = 0.399 ± 0.031; *N* = 172) to a high in the Antarctic (*π* = 0.0043 ± 0.0005; *h* = 0.622 ± 0.026; *N* = 137; table 2). These values are comparable with those for various marine mammal species known to have been through a population bottleneck (see Hoelzel, Goldsworthy, et al. 2002). An earlier study reported evidence for population expansion for a global sample set that excluded southern Africa (Hoelzel, Natoli, et al. 2002), but there was no evidence for expansion from the South African sample provided here (Tajima’s *D* = 0.973; *P* = 0.87; Fu’s *F*_s_ = −1.526, *P* = 0.285). Significant values for either Tajima’s *D* or Fu’s *F*_s_ suggest a deviation from neutrality and either strong selection or a postbottleneck expansion. Our analysis using 5,043 bp from the published mitogenomes (providing greater resolution for tests of historical demographics; see Materials and Methods) suggested evidence for a spatial expansion that dates to 889 or 8,892 years before present (applying the fast and slow rate, respectively, see Materials and Methods; mismatch distribution: sum of squared deviations [SSD] = 0.012, *P* = 0.33, *τ* = 1.78; Fu’s *F*_s_ = −24.21, *P* < 0.00001, Tajima’s *D* = 0.095, *P* = 0.61) and a numerical expansion starting at 11.3 ka (thousand years ago) or 113 ka (SSD = 0.007, *P* = 0.76, *τ* = 22.8; where SSD is the sum of squared deviations, used as a test statistic comparing the observed and expected mismatch values, and *τ* is a moment estimator of the time of expansion). Note that the nonsignificant *P* values for SSD indicate that the observed distribution did not deviate significantly from the model distribution for expansion (Rogers et al. 1996). The 95% confidence limits for the estimate of *τ* were broader for the spatial expansion (0.34–132.46) than for the numerical expansion (1.19–32.65).

**Table 2.** mtDNA Diversity Measures for Comparative Sample Sets from Different Geographic Regions.

Population	Number of Haplotypes (*k*)	*h* ± S.D.	*π* ± S.D.	*N*	*k*/*N*
South Africa	13	0.889 ± 0.030	0.0147 ± 0.0008	37	0.351
Antarctic	9	0.622 ± 0.026	0.0043 ± 0.0005	137	0.065
North Atlantic	8	0.483 ± 0.035	0.0019 ± 0.0002	136	0.059
North Pacific transients	6	0.350 ± 0.050	0.0021 ± 0.0005	134	0.045
North Pacific residents	2	0.399 ± 0.031	0.0013 ± 0.0001	172	0.012

Note.—*h* is haplotype diversity, *π* is nucleotide diversity and *N* is the sample size.

## Discussion

Modern killer whale populations show strong geographic differentiation, and low genetic diversity within regional matrilineal populations (Hoelzel, Natoli, et al. 2002; Hoelzel et al. 2007; Morin et al. 2010). Differentiation is sometimes correlated with ecotype specializations (e.g., consistently feeding on marine mammal vs. fish prey; Hoelzel, Dahlheim, et al. 1998; Morin et al. 2010), though not exclusively (LeDuc et al. 2008; Morin et al. 2010). There is some discontinuity between phylogeography based on mtDNA and nuclear DNA markers (Hoelzel et al. 2007; Pilot et al. 2010), previously suggested to be due to a stochastic distribution of haplotypes following an historical bottleneck event (Hoelzel, Natoli, et al. 2002). A study based on whole mtDNA genome sequences identified three main phylogenetic lineages representing 2–4 ecotypes per lineage (Morin et al. 2010); however, it also revealed a remarkably low level of diversity with the most distinct haplotypes differentiated by only 0.56%, and some individuals on either side of the North Pacific differing by only 1 bp out of the full mitogenome sequence (comparing the Russian Kamchatka population with the Washington State southern residents). The time to most recent common ancestor (TMRCA) for the mitogenome tree (Morin et al. 2010) was estimated at 700 ka based on a conservative mutation rate estimate (an order of magnitude slower than an independent estimate for cetacean mitogenomes; Ho and Lanfear 2010). Comparing 64 mitogenome sequences representing most major populations (Morin et al. 2010), *π* is less than 0.005 for over 90% of the sequence (supplementary fig. S3, Supplementary Material online). Taken together, the data on modern killer whale population genetics and phylogeography from a broad range of locations across the world are consistent with historical bottlenecks leading to the loss of diversity. Based on our mtDNA data, the South African population stands out as an exception showing an order of magnitude greater diversity than for other regions (table 2; see further discussion later). Our nuclear genome analysis showing a relatively high Ne inferred around the time of the Eemian interglacial, together with mtDNA data indicating an expansion into the Holocene, suggests that the decline was not associated with global warming but instead with the glacial period following the Eemian.

Apex predators are sensitive to disturbances in prey resources, which may be impacted by rapid climate change (see review in Introduction). Alternative explanations for population decline include epizootics and exploitation, and it is likely that the factors affecting killer whale population dynamics and distribution are overlapping and complex. However, known cetacean epizootics have been on a regional scale (Lipscomb et al. 1996), and the decline is too ancient for credible anthropogenic exploitation. Although early whaling may date back to as far as 6,000 BCE (possibly including takes of killer whales [Lee and Robineau 2004]), logistically killer whale hunting by Paleolithic humans could not have occurred on a sufficient scale to promote the global decline of an oceanic animal. Although the impact of Pleistocene climate cycles was global, it also varied among regions due, for example, to variation in the Earth’s land–sea distribution (Short et al. 1991) as indicated in the regional variation of signals from biotic indicators (e.g., fig. 4 in Webb and Bartlein 1992). At the same time, data showing similar demographic profiles for killer whale populations in the North Pacific and North Atlantic suggests a shared history or at least historical connectivity among these regions (fig. 1), despite significant differentiation at microsatellite DNA markers among the modern populations (Hoelzel et al. 2007). While this shared impact may reflect climate change, there is an apparent lack of impact from earlier glacial cycles (also shared), and a possible increase during the Saalian glacial that preceded the Eemian interglacial (in the trace for the North Pacific). Therefore, any causal explanation associated with glaciation would need to account for this discontinuous association with the longer term climate cycles.

However, some data do distinguish the Weichselian (most recent) glacial period, characterized by the lowest recorded temperature (at the LGM) and a more unstable climate compared to the rest of the Pleistocene cycles (Berger 2008). Given the dependence of apex predators on an abundant prey resource (see Noren [2011] for a review of studies on killer whale energetic requirements), a candidate for a causative environmental factor would be changes in ocean productivity. Various studies have described changes in marine paleoproductivity during the Quaternary climatic oscillations (see Berger et al. 1989). Sediment core data from the North Atlantic (NW Africa) based on high resolution proxy environmental measures indicated that the LGM had warmer sea surface temperature due to weaker upwelling (associated with the Canaries upwelling system), and lower marine productivity compared with earlier Pleistocene glacial and interglacial periods (Zhao et al. 2000). Increased levels of denitrification during the Eemian interglacial may have caused a negative net nitrogen balance in the oceans leading to a decline in productivity during the Weichselian (Xing et al. 2011), though higher levels of nitrogen fixation may have been compensatory (Ren et al. 2009). However, greater productivity likely persisted in the Southern Ocean due in part to the introduction of iron from glacial dust (Francois et al. 1997; Latimer and Filippelli 2001).

Upwelling enriches coastal productivity at numerous locations around the world, and although there is no strict dependency between upwelling and modern killer whale distribution, these systems generate sufficient biomass to support top predators. There is also no way to know if current prey choice reflects historical behavior for a given population, but killer whales forage at high trophic levels in all modern populations. At present, there are four predominant upwelling systems in the world which are well described in the literature, two in the Northern Hemisphere and two in the Southern Hemisphere. The Benguela cold-water current, which today supports a rich upwelling system and very high marine productivity off southern Africa, was established ∼10 Ma and intensified at the Pliocene–Pleistocene climate transition, with the strongest upwelling developing by ∼1 Ma and persisting to the present (Marlow et al. 2000; Etourneau et al. 2009; Heinrich et al. 2011). This system interacts with the Agulhas current and supports a diverse local ecosystem including the dense annual sardine (*Sardinops sagax*) run and associated predators (including known killer whale prey species) along the South African eastern coast (Fréon et al. 2010). Although there is some indication of fluctuations in the Benguela system, possibly in association with leakage of warm water from the Agulhas into the Atlantic during interglacials, upwelling likely remained strong (Lazarus et al. 2008).

The other major system in the Southern Hemisphere is the Humboldt current in the Pacific off Peru and Chile, and it presently represents one of the most productive marine regions in the world (Montecino and Lange 2009). This system was also likely present through the Pleistocene (Mohtadi and Hebbeln 2004); however, over the last 5,000–7,000 years productivity has been regularly disrupted by the El Nino cycles (Montecino and Lange 2009), which are known to have a disruptive influence on local marine species (Riascos et al. 2009). Although modern day sightings of killer whales are common off southern Africa, indicative of an abundant local population (Best et al. 2010), sightings are rare off Peru (García-Godos 2004).

In the Northern Hemisphere, the two major systems are the Canary current and the California current. As described earlier, there are indications of a reduction in upwelling activities in regions associated with the Canary current during the LGM (Zhao et al. 2000). Climate records and sea surface temperature estimates indicate that the other major system, the California current, collapsed during the glacial maxima phases of the Milankovitch cycles (Herbert 2001). Therefore, among the four main upwelling regions worldwide (Benguela off South Africa, Humboldt off South America, the Canaries system off Northern Africa and the California current off North America) only the Benguela system appears to have remained consistently productive since the Eemiam interglacial. At the same time, high latitude coastal regions in the Southern Ocean off New Zealand and eastern South America are also thought to have acted as refugia for some marine species during the Pleistocene glacial cycles (Fraser et al. 2009, 2012). Preliminary studies indicated low killer whale diversity in these regions (Hoelzel, Natoli, et al. 2002), but further research is needed to confirm that the South African population is the only one to retain high levels of diversity.

Our data on mtDNA diversity, synthesized from some new and extensive published data, show low worldwide diversity in all investigated regions apart from southern Africa (fig. 3). This supports earlier interpretations suggesting a population bottleneck (Hoelzel, Natoli, et al. 2002), though data are not evenly distributed, and greater diversity in the Southern compared with the Northern Hemisphere is possible. Even so, the southern African population in particular is disproportionately diverse (e.g., 3–4 times higher gene diversity than in the Antarctic; table 2), suggesting that it represents a regional refuge not impacted by a loss of diversity elsewhere. A possible alternative explanation may be the convergence of whales into the southern African region from diverse populations in the recent past. However, the low worldwide level of mtDNA diversity, the distribution of southern African haplotypes in the center of the network reconstruction, and the presence of private alleles in the southern African sample, are all more consistent with a stable regional population.

When haplotypes from all other sampled regions are combined (including both Southern and Northern Hemisphere regions), there is a strong expansion signal dating to after the LGM (for a spatial expansion regardless of the mutation rate, and for a demographic expansion using the higher rate; see Materials and Methods). Our results based on nuclear data reflecting historical trends in the Northern Hemisphere indicate that events concurrent with the last (Weichselian) glacial period induced a severe population decline in this top marine predator, unique for this species during the Pleistocene timeframe. Therefore, both nuclear and mtDNA data are consistent with respect to the severity and timing of a bottleneck event (though the confidence limits are much tighter for the estimates based on the nuclear data). We cannot easily distinguish between a model of expansion from a single refuge and the founding of regional populations, compared with the decline of populations in most locations, some more extensively than others, or some combination of these processes. However, our mtDNA data are consistent with a postbottleneck expansion worldwide, with the exception of South Africa (where reduced historical population size remains possible, but not detected with the available data). Additional genomic data may in future provide further resolution.

Although we cannot exclude alternatives associated with some unknown epizootic, or independent factors associated with climate change (such as the redistribution of prey), the association between the one known population retaining diversity and a stable productive environment suggests that ocean productivity may have been a key factor. Species that track changing habitat in the marine environment by redistribution can apparently remain abundant through climatic transitions (Foote et al. 2013). Ecological shifts in particular may have allowed killer whales to do the same. Our data suggest instead a decline in most regions of the species range (inferred for the Northern Hemisphere based on the nuclear genomes, and for all sampled regions except southern Africa based on the mtDNA data). If reduced productivity is a causative factor, this would emphasize the importance of stable productive systems (such as the waters off southern Africa) during periods of environmental change. The decline of the killer whale suggests substantial changes in marine ecosystems correlated with climate change, and the possibility of a similar impact on other marine apex predators.

## Materials and Methods

### DNA Extraction and Genomic Sequencing

Whole-genomic DNA was extracted from a male individual from Southeast Alaska (from the fish-eating ecotype Alaskan resident population) using standard protocols. Whole-genome sequencing was done at Eurofins MWG Operon (Ebersberg, Germany). A short insert shotgun library (SG library) was based on protocols from the Illumina manual. In brief, 3.5 µg of DNA was fragmented using a Covaris E210 Instrument (Covaris Inc., Woburn, MA) according to manufacturer’s instructions, followed by end repair, A-tailing, and ligation of the indexed Illumina adapter. Ligation products were size selected by agarose gel, targeting an average insert size of 250 bp. After polymerase chain reaction (PCR) amplification the resulting fragments were cleaned up and used for cluster generation. An additional 8-kb mate-pair-like long jumping distance (LJD) library was prepared based on the mate-pair library protocol from Illumina, modified by using adaptor-guided ligation of genomic fragments which achieves higher accuracies.

For sequencing both libraries, cluster generation was performed using manufacturer’s instructions. Paired-end sequencing was performed on a HiSeq2000 machine (HiSeq Control Software 1.5.15.1) using HiSeq Flow Cell v3 and TruSeq SBS Kit v3. For processing of raw data RTA version 1.13.48.0 and CASAVA 1.8.2 was used to generate FASTQ-files. From the SG library, 52.364 Gb were obtained with a Q30 of 84.83% and 89.76% of reads passing the Illumina default filter (chastity filter). For the LJD library, 12.758 Gb were obtained with a Q30 of 76.33% and 87.12% of reads passing the default filter.

### Short-Read Mapping

Illumina short reads were cleaned from adapter sequences and trimmed from low quality bases at the end of reads using the software Trimmomatic version 0.2 (Lohse et al. 2012), with a length-cutoff of 30 bp (MINLEN), and arguments-threads 20-phred 33. After cleaning and trimming, 5.33% of SG reads and 9.93% of LJD reads were dropped, while 10.59% of SG and 37.38% of the LJD reads were singletons. Trimmomatic parameters LEADING and TRAILING were set to the quality score threshold of 20.

Whole-genomic sequences where assembled by mapping cleaned and trimmed short reads to a reference genome using BWA version 0.6.2 (Li and Durbin 2009). As a reference, we used the bottlenose dolphin (*Tursiops truncatus*) genome build 1.69 retrieved from the Ensembl database. This represents the best annotated version of a cetacean genome available, and given the stability of cetacean karyotype (Arnason 1982), and the close phylogenetic relationship among delphinids (maximum estimated age for the most recent common ancestor ∼10 Ma; Steeman et al. 2009), it represents the most appropriate reference for short read mapping of the killer whale. The X chromosome was removed from the bottlenose reference genome prior to mapping of the killer whale short reads. This was done by mapping all the scaffolds from the bottlenose dolphin build 1.69 mapped against the X chromosome from the cow genome build 3.1.69 (retrieved from the Ensembl database), using the mapping application in the software package Geneious with medium sensitivity. Both genomes were retrieved from the Ensembl website. From the initial 111,212 scaffolds, 1,620 matching scaffolds were removed from the genome build to create a pseudoautosomal reference genome, against which killer whale short reads were mapped. Both paired end libraries (SG and LJD) and corresponding singletons were mapped independently, with resulting bam files merged in SamTools (Li et al. 2009) version 0.1.18. Genotypes at each mapped site were called using the SamTools mpileup option, with the coefficient for reducing mapping quality for reads containing excessive mismatches set to 50 (following suggestion from the software manual), and Bayesian inference using bcftools, with the output stored as a vcf file. Average depth coverage was calculated using vcfTools version 0.1.9 (Danecek et al. 2011), and repetitive regions where heterozygosity might be artificially inflated due to excessive read mapping were discarded by removing all sites with more than twice the average coverage in subsequent analysis.

The adequacy of this approach was further assessed by analysing read coverage across the genome using Savant Genome Browser (Fiume et al. 2010). All positions with coverage below 4 were removed. The output vcf file from SamTools mpileup/bcftools genotype calling was converted into FASTQ using the vcfutils perl script from the SamTools package, which was then loaded into Geneious software package (Drummond et al. 2010) for visualization and calculation of basic statistics such as the number of total bases called and total number of homozygous vs. heterozygous positions. The resulting sequence was 2.23 Gb in length with 41.25% GC content and 0.0434% heterozygote positions. Average heterozygosity was calculated in 50 kb windows using vcfTools nucleotide diversity option, with sites exhibiting mapping quality below 20 removed. We then excluded all sites with coverage higher than twice the average, and lower than 4×. The 50-kb bins were then derived only from those sites that passed these filters, and Ht counted from among those sites. The normalized probability distribution histogram of heterozygosity per 50-kb window was calculated using R version 2.14.0. The same procedure was repeated for a subsample of the database (North Atlantic) killer whale reads (truncated from downloaded files; GenBank: SRA058929) so that the analyses would be based on a comparable level of resolution. Resulting genomic sequences were confirmed to be representative, comprising 2.23 Gb in length (representing 96.97% of the reference genome) with 20× average depth of coverage for the North Pacific killer whale, and 2.15 Gb in length (93.45% of the reference) and 13× coverage for the North Atlantic killer whale.

For comparison, we calculated genomic heterozygosity for the Chinese rhesus monkey (*M. mulatta*) and the database killer whale (thought to be from a fish eating population in the North Atlantic) sequence using the same approach. Short reads for the rhesus monkey were retrieved from the NCBI short read archive (GenBank: SRA037810 and SRA023854) and mapped to its reference genome (version 1.69 retrieved from the Ensembl database; Fang et al. 2011) using the same approach described earlier. Heterozygosity for the monkey genome was calculated as described earlier, with a minimum filter of 4-fold coverage, resulting in an average actual coverage of 6× for the monkey, 13× for the North Atlantic killer whale and 20× for the North Pacific killer whale. Normalized probability distribution histograms of heterozygosity were carried out as described earlier.

### Demographic Analysis

Analysis of demographic history for both North Pacific and North Atlantic genomic sequences (obtained as described earlier) was done using the PSMC model, as implemented in the PSMC software package (Li and Durbin 2011). We chose this method because it is now well represented in the literature, shows good correspondence to expected patterns of demography for various species, and good reproducibility among individuals from a given species or population (Prado-Martinez et al. 2013). This method derives the demographic profile from reconstructing the TMRCA distribution across autosomes (such that one or a few individual genomes reflect the demography of whole populations, regions or species), and the authors demonstrate the utility of this approach both by comparing genomes from different individuals (and showing that the profiles match), and through simulation analyses—simulating one hundred 30-Mb sequences with shared demography (Li and Durbin 2011). Li and Durbin (2011) also show from simulations that an instantaneous change would be seen as a more gradual event. All positions with mapping quality below 20 were filtered out when converting from the FASTQ file to the PSMC 100-bp bin input file. Time parameters were set to 4 + 25 × 2 + 4 + 6 as suggested in (Li and Durbin 2011). Robustness of demographic estimates was assessed by bootstrapping with 100 iterations, as described in Li and Durbin (2011). Generation time for real-time scaling was set to 25.7 (see Taylor et al. [2007] for discussion on uncertainties of parameter estimates).

The calculation of mutation rates shows some rate variation among lineages for cetaceans (Dornburg et al. 2012), with estimated mutation rates for mysticetes typically being lower than for odontocetes (Jackson et al. 2009; Dornburg et al. 2012). However, we use an average of the total published cetacean range together with the two extremes (Alter et al. 2007; Jackson et al. 2009; Dornburg et al. 2012; McGowen et al. 2012). The resulting average mutation rate was 1.53 × 10^−^^8^ substitution/ nucleotide/generation. To assess the effect that using different mutation rates would have on our interpretations, we rescaled using a faster rate calculated for only odontocetes of 2.34 × 10^−^^8^ (Dornburg et al. 2012), and a slower rate calculated using only mysticetes of 1.05 × 10^−^^8^ (Jackson et al. 2009).

### mtDNA Analyses

Our strategy for mtDNA analyses was to incorporate our data into as much of the extensive published database as possible, and to use a highly diverse marker for the comparison of global diversity, together with a longer sequence derived from the noncoding regions of published mitogenomes for demographic inference. A sliding window of 300 bp incremented by 10-bp steps implemented in DnaSP (Librado and Rozas 2009) identified the most variable region within the killer whale mtDNA genome (based on 65 haplotypes [Morin et al. 2010]; supplementary fig. S3, Supplementary Material online), and the South African samples were sequenced for this fragment. The control region showed the highest level of diversity, and the identified 300-bp segment was within the 5′ end of that locus. For changes present in more than one individual, there were ten sites within the 300 bp segment, but only four for the remainder of the control region (indicating that the chosen sequence provided most of the informative sites within the control region). A focus on the control region permitted the comparison of 616 samples including available published data. Samples sequenced for this study include five from Iceland, two from Tierra del Fuego and one from Japan (sequenced for the full mtDNA control region using protocols described in Hoelzel et al. [2007]), and 37 from South Africa. The South African samples were amplified for the 300-bp fragment in one or two (for nine samples) segments as necessary. These samples were from museum collections and generally of poor quality (necessitating the focus on a relatively short fragment). Details of sample dates and locations together with museum source are provided in supplementary table S2, Supplementary Material online.

The primers used were as follows: F-5′ TCGATTATATCCTATGGT 3′ and R-5′ ATGAAAAATACACACAGG 3′ (Ta = 49 °C) for samples sequenced in one fragment (amplifying 336 bp including primers). For samples sequenced in two overlapping approximately 200-bp fragments the primer sets used were as follows: F-5′ TCGATTATATCCTATGGT 3′ and R- 5′ TTTATGGGCTGATTAGTC 3′ (*T*_a_ = 49 °C), F- 5′ CCTTGCCTAACATAACTG 3′ and R- 5′ ATGAAAAATACACACAGG 3′ (*T*_a_ = 49 °C). The PCR amplification cycle was as follows: Hot start of 95 °C for 15 min and 45 cycles of denaturation at 94 °C for 30 s, annealing at respective Ta for 90 s and elongation at 72 °C for 1 min. This was followed by an elongation step of 72 °C for 10 min.

DNA extraction from tooth and bone samples (supplementary table S2, Supplementary Material online) was performed according to the following protocol: DNA extractions and PCR setup were conducted in a laboratory dedicated exclusively to ancient DNA analysis, physically separated from other laboratories. Contamination was monitored by using negative controls for both extraction and PCR procedures. About 100 mg of bone powder was digested in 1 ml of extraction buffer, containing 0.4% sodium dodecyl sulphate, 0.5 M ethylenediaminetetraacetic acid (pH 8), and 30 µl proteinase K (20 mg/ml), at 37 °C overnight, followed by 1 h in 50 °C. Digests were centrifuged at 13,000 × g for 1 min. Further steps of the extraction procedure were performed using QIAquick PCR Purification Kit (Qiagen) following the manufacturer’s instructions.

Independent extractions were undertaken for three samples and sequenced to confirm genotypes. All PCR fragments were sequenced in both directions and sequencing replicated for a given extract 1 to 5 times. All repeat sequences as well as forward and reverse pairs were checked manually to ensure consistency. Available information on provenance and museum sampling ruled out the possibility of unknowingly sequencing replicate samples from the same individual (supplementary table S2, Supplementary Material online). The individual haplotype names (supplementary table S2, Supplementary Material online) and all accession numbers are provided in supplementary table S1, Supplementary Material online, and the haplotype numbers identified in the network in figure 2. Diversity and metrics to assess expansion signals were assessed using Arlequin (Excoffier et al. 2005) and median joining networks constructed using the program Network 4.611 (http://www.fluxus-engineering.com/sharenet.htm, last accessed February 14, 2014). Sequences generated during this study were compared against the homologous sequence from published data (Hoelzel, Dahlheim, et al. 1998; Barrett-Lennard 2000; Hoelzel, Natoli, et al. 2002; Zerbini et al. 2006; Hoelzel et al. 2007; Pitman et al. 2007; LeDuc et al. 2008; Morin et al. 2010; Foote et al. 2011). Given a worldwide distribution of samples, to improve resolution and accuracy, we applied a mismatch distribution analysis to 5,043 bp (excluding protein coding genes) published data from mitochondrial DNA genomes (Morin et al. 2010) representing haplotypes from Antarctica, the South Pacific, North Pacific and North Atlantic. As sampling by region was uneven, and some populations reflect extended matrilines of close kin, we included only unique haplotypes (after Hoelzel, Natoli, et al. 2002). A generation time of 25.7 years (Taylor et al. 2007) was applied and two mutation rates: 2%/million years (after Ho and Lanfear 2010) and an order of magnitude higher rate (20%/million years) reflecting the time dependency of rates discussed in Ho et al. (2005).

## Supplementary Material

Supplementary figures S1–S3 and tables S1 and S2 are available at *Molecular Biology and Evolution* online (http://www.mbe.oxfordjournals.org/).

Supplementary Data
