# Supplementary material for: Killer Whale Nuclear Genome and mtDNA Reveal Widespread Population Bottleneck during the Last Glacial Maximum
Source: Mol Biol Evol. 2014 Feb 4;31(5):1121–31. doi: 10.1093/molbev/msu058 (PMC3995335; doi:10.1093/molbev/msu058)
Supplement: Supplementary Data [file supp_31_5_1121__index.html]

Killer Whale Nuclear Genome and mtDNA Reveal Widespread Population Bottleneck during the Last Glacial Maximum — Killer Whale Nuclear Genome and mtDNA Reveal Widespread Population Bottleneck during the Last Glacial Maximum — Supplementary Data 

# Killer Whale Nuclear Genome and mtDNA Reveal Widespread Population Bottleneck during the Last Glacial Maximum

## Supplementary Data

files

**Files in this Data Supplement:**

- Supplementary Data - docx file
