# Supplementary material for: Killer Whale Nuclear Genome and mtDNA Reveal Widespread Population Bottleneck during the Last Glacial Maximum
Source: Mol Biol Evol. 2014 Feb 4;31(5):1121–31. doi: 10.1093/molbev/msu058 (PMC3995335; doi:10.1093/molbev/msu058)
Supplement: Supplementary Data [file supp_msu058_Moura_et_al_supp_MBE_24Jan14.docx]

**Moura et al. Supplementary File**


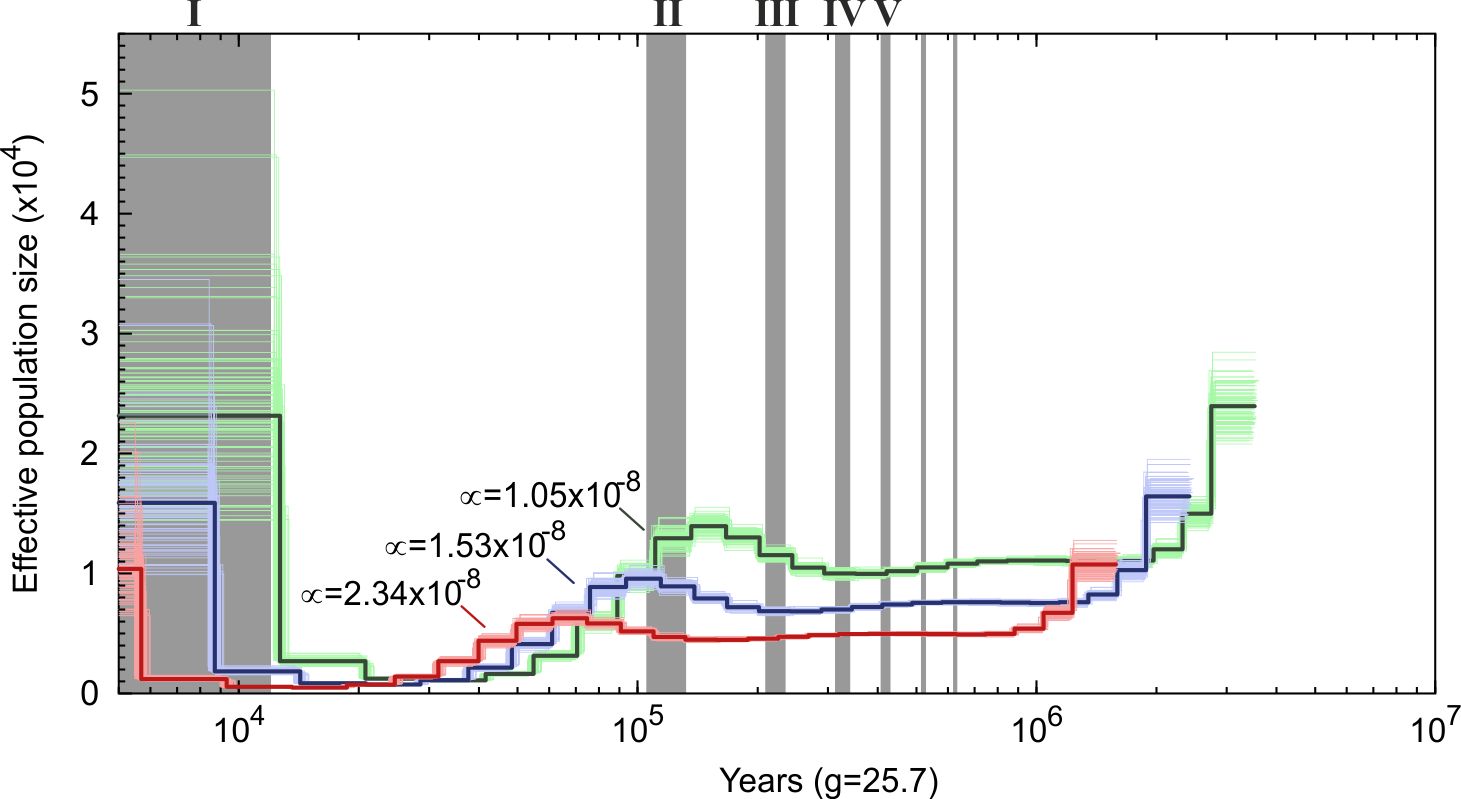


**Figure S1:** PSMC analyses for North Pacific killer whale based on varying estimates of the substitution rate.


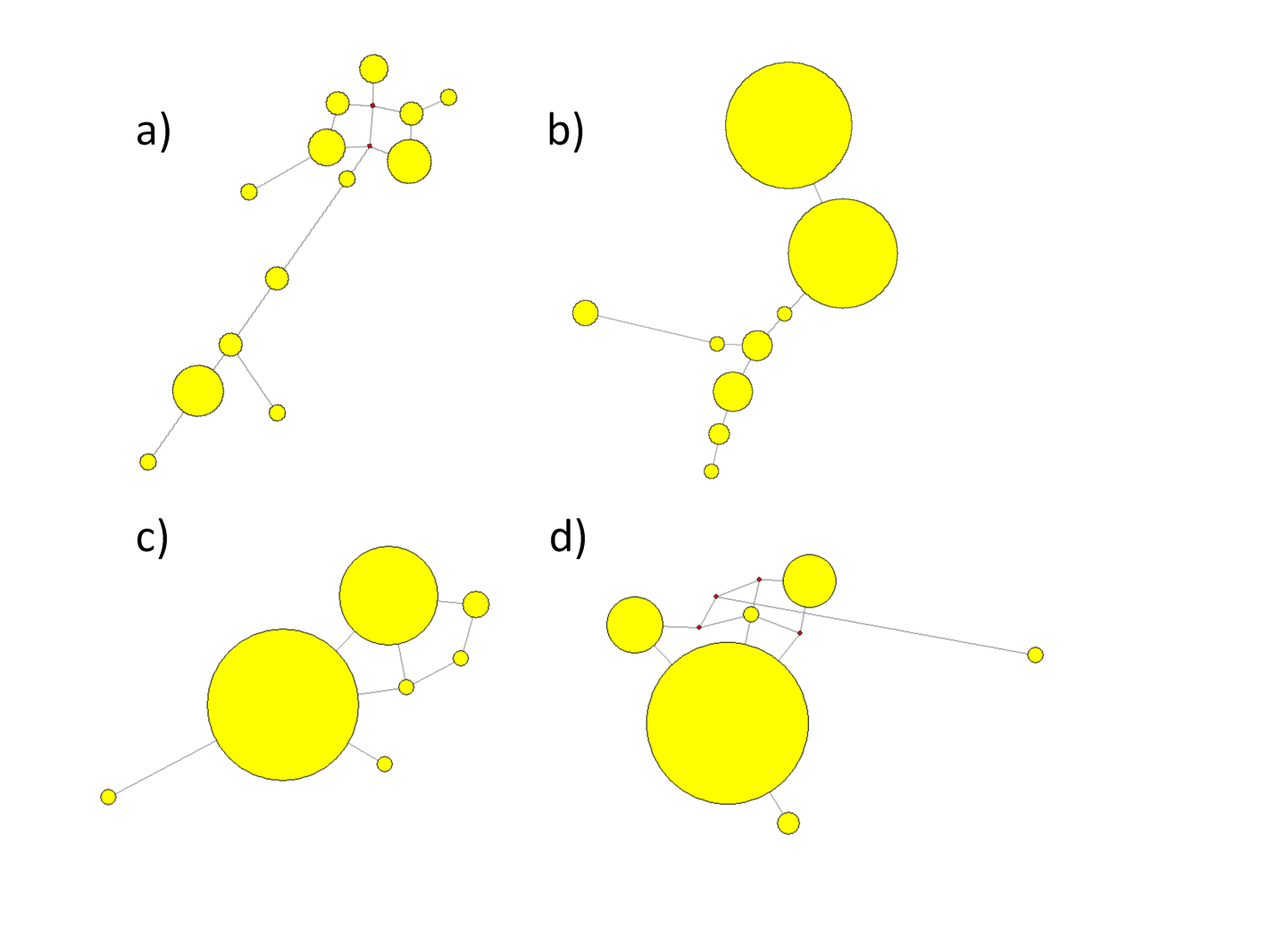


**Figure S2.** Median spanning networks for samples from a) South Africa, b) the Antarctic, c) the North Atlantic, and d) the ‘transient’ (marine mammal eating) ecotype in the North Pacific. Circle size reflects the relative number of individuals for a given haplotype. The North Pacific resident populations are not shown because there are only two haplotypes at this locus (differentiated by 1bp), one in 47 individuals, and the other in 125.


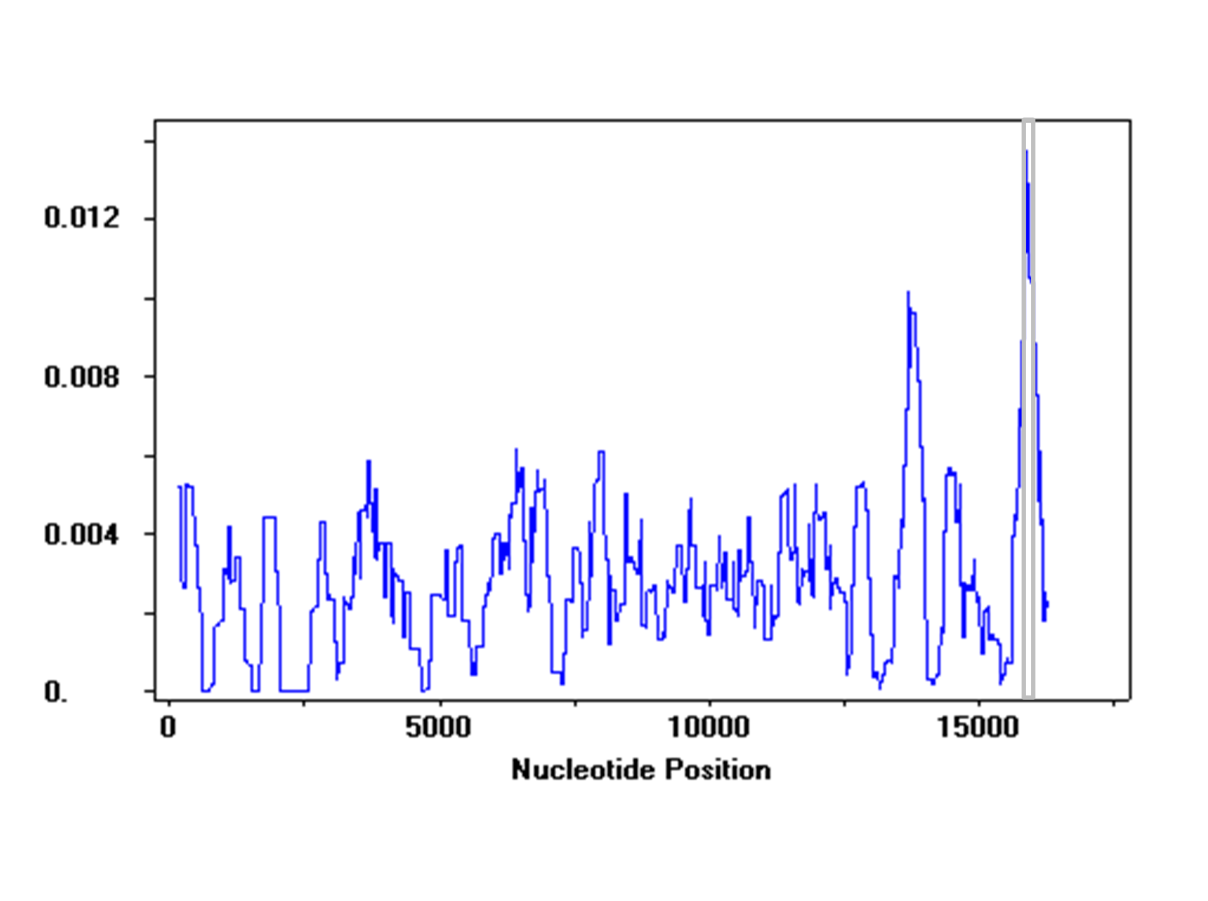


**Figure S3:** Estimates of π based on a sliding window analysis across 67 published killer whale mitochondrial genomes. Grey box shows sequence chosen for comparative analyses.

**Table S1:** Haplotypes by name (published names after references cited in methods text), NCBI accession codes (in the same sequence as the names) and numbers (from Figure 2 in the main document). Most name codes follow the convention whereby E=eastern, W=western, C=central, N=north, S=south, T=tropical, A (or Atl)=Atlantic, P=Pacific, and ANT (or Ant)= Antarctic. SAB= a South African sample from bone or tooth. SAT= a South African sample from tissue. The remaining codes, NEWR, NT1, NT2 and GAT are from the ENP. Sequences without accession codes can be found by the name code in [1,2].

| Hap. | Haplotype names | Accession code |
| --- | --- | --- |
| 1 | NC_014682; ENAC2; WNAUCAN; ENPOAL1; ETPUMex2; WNPNRRU; CNPNRAL1; ENAUI_ENAME; ETPUMex_C1; ENPOGA; ENPSRBC; CNPNRAL2; ENAUS; ENPOAL1; WNPNRAL; ANT8; ENPSR; ENPO; NEWR; ETP1; ENAUK2; ENAC1; ENAG2; ENAS2; Atl_2_28; ANTRS | NC_014682; HQ405752; GU187202; GU187201; GU187198; GU187196; GU187190; GU187188; GU187199; GU187197; GU187195; GU187191; GU187154; GU187192; GU187200; EU714130; DQ399077; DQ399079; DQ399074; DQ851147; GQ303362; HQ405753; HQ405755; HQ405754; GQ303363 |
| 2 | AntA4; ANT5 | GU187219; EU714127 |
| 3 | AntA1; AntA3; ANT4; ANT3; WSAPN; SAB1; SAB3; SAB5; SAB32; SAB35 | GU187217; GU187218; EU714126; EU714125; KJ185106 |
| 4 | AntB1; AntB2; AntC1; AntC5; AntC2; AntB4; AntB3; AntC10; AntC3; ANT13; ANT12; ANT11; ANT10; SAB4; SAB7; SAB44 | GU187215; GU187213; GU187210; GU187208; GU187211; GU187214; GU187212; GU187209; GU187207; EU714135; EU714134; EU714133; EU714132; KJ185107 |
| 5 | AntC9; AntC8; AntC7; AntC4; AntC6; ANT15; ANT14 | GU187206; GU187204; GU187153; GU187205; GU187203; EU714137; EU714136 |
| 6 | ENPNRAL2; ENPNRGA_AL_PI_ENPUCA; CNPNRAL; ENPAR | GU187194; GU187193; GU187189; DQ399078 |
| 7 | ENAHN5; ENAHN6; ENAHN3; ENAHN2; ENAHN8; Atl_1_35 | GU187186; GU187184; GU187182; GU187183; GU187181; GQ303370 |
| 8 | ENAHI1; ENAHN1; ENATG; ENAHI2; ENAHN4; SWPUNZ; ENAI1_ENAUK1; ENAI2_WSAPNZ1; SAB28; SAB31; SAB33; SAB36L; SAT38; SAT39; SAB42; Atl_1_31; Atl_1_32; Atl_u_36; WSPNZ2; WSANA | GU187180; GU187178; GU187176; GU187179; GU187177; GU187175; GQ303369; KJ185117; GQ303366; GQ303367; GQ303371 |
| 9 | ENPTGA2; WNPTAL1_CNPTAL; CNPUBS; ENPTCA_ENPUCA2; ENPUCA4; WNPTRU1; ENPTAL2; ENPTAL3; ENPTPI1; ENPUCA3; ENPTSEA2; ENPTPI2; NT2; GAT; NT1_WNPJ; ENPT1; ENPT3 | GU187174; GU187172; GU187170; GU187168; GU187163; GU187159; GU187173; GU187171; GU187169; GU187167; GU187162; GU187160; DQ399076; DQ399081; DQ399075; KJ185119 |
| 10 | ETPUHI2; SAB11; SAB16 | GU187166; KJ185111 |
| 11 | WNPTRU3 | GU187161 |
| 12 | WNPTRU4; WNPTAL2; WNPTRU2; ENPT5_AT1 | GU187157; GU187158; GU187156; DQ399082 |
| 13 | AntA2; ENPTAL1; ALHGAT2; ANT2; ANT1; ENPT2; SAB23; SAB24 | GU187155; GU187164; DQ399080; EU714124; EU714123; DQ399080; KJ185114 |
| 14 | WNAUGM; WNA1; WTAGM | GU187216; DQ845456; GQ303372 |
| 15 | ETPUHI1; ANT6; SAB40 | GU187187; EU714128; KJ185118 |
| 16 | ENAHN7 | GU187185 |
| 17 | WNPK | EF635420 |
| 18 | ANT9 | EU714131 |
| 19 | ANT7 | EU714129 |
| 20 | ETP2 | DQ851148 |
| 21 | ENAI3 | KJ185122 |
| 22 | ENAUK3 | KJ185123 |
| 23 | ENPT4 | KJ185120 |
| 24 | SAB6 | KJ185108 |
| 25 | SAB8; SAB9; SAB12; SAB13; SAB15; SAB18; SAB19; SAB21; SAB45 | KJ185109 |
| 26 | SAB10 | KJ185110 |
| 27 | SAB17 | KJ185112 |
| 28 | SAB22; SAB34 | KJ185113 |
| 29 | SAB25; SAB26 | KJ185115 |
| 30 | SAB27 | KJ185116 |
| 31 | Atl_1_30 | GQ303365 |

1. Hoelzel AR, Natoli A, Dahlheim ME, Olavarria C, Baird RW, Black NA: **Low worldwide genetic diversity in the killer whale (*Orcinus orca*): implications for demographic history**. *P Roy Soc Lond B Biol* 2002, **269**:1467–1473.

2. LeDuc RG, Robertson KM, Pitman RL: **Mitochondrial sequence divergence among Antarctic killer whale ecotypes is consistent with multiple species**. *Biol Lett* 2008, **4**:426-429.

**Table S2:** Sample codes and details for material collected in South Africa. ISAM= Iziko South African Museum; DOE= Department of Environment Affairs; PEM= Port Elizabeth Museum.

| ID | Sex | Year | Locality | Tissue | Source |
| --- | --- | --- | --- | --- | --- |
| SAB1 | F | 1971 | near Durban | Tooth | ISAM |
| SAB1 duplicate |  |  |  |  |  |
| SAB3 | M | 1971 | near Durban | Tooth | ISAM |
| SAB4 | M | 1972 | near Durban | Tooth | ISAM |
| SAB5 | F | 1971 | near Durban | Tooth | ISAM |
| SAB6 | M | 1971 | near Durban | Tooth | ISAM |
| SAB7 | M | 1972 | near Durban | Tooth | ISAM |
| SAB8 | M | 1972 | near Durban | Tooth | ISAM |
| SAB9 | F | 1972 | near Durban | Tooth | ISAM |
| SAB10 | M | 1972 | near Durban | Tooth | ISAM |
| SAB11 | M | 1972 | near Durban | Tooth | ISAM |
| SAB12 | F | 1972 | near Durban | Tooth | ISAM |
| SAN13 | M | 1972 | near Durban | Tooth | ISAM |
| SAB15 | M | 1972 | near Durban | Tooth | ISAM |
| SAB16 | M | 1972 | near Durban | Tooth | ISAM |
| SAB17 | M | 1973 | near Durban | Tooth | ISAM |
| SAB18 | M | 1973 | near Durban | Tooth | ISAM |
| SAB19 | M | 1973 | near Durban | Tooth | ISAM |
| SAB21 | F | 1973 | near Durban | Tooth | ISAM |
| SAB22 | F | 1963 | near Saldanha Bay | Tooth | ISAM |
| SAB23 | M | 1975 | near Durban | Tooth | ISAM |
| SAB24 | F | 1975 | near Durban | Tooth | ISAM |
| SAB25 | M | 1969 | Bordjiesdrif, Cape Point Nature Reserve | Tooth | ISAM |
| SAB26 | F | 1968 | Blouberg Beach, Cape Town | Tooth | ISAM |
| SAB27 | M | 1974 | 3104S 5850E | Tooth | ISAM |
| SAB28 | M | 1991 | Danger Bay, Saldanha | Tooth | ISAM |
| SAB31 | M | ~1915 | Off Saldanha Bay | Bone | ISAM |
| SAB32 | M | 1963 | Kreefte Baai, Churchaven | Bone | ISAM |
| SAB33 | U | U | Found un-numbered in collection | Bone | ISAM |
| SAB34 | M? | 1980 | Riebeeksdam, Cape Point Nature Reserve | Tooth | ISAM |
| SAB35 | M | 1999 | West Point, St Helena Bay | Tooth | ISAM |
| SAB36 | U | 2010 | 34 17.5S 17 38.8E | Bone | ISAM |
| SAT38 | F | 2010 | Melkbosstrand, N of Cape Town | Skin | DOE |
| SAT39 | F | 2009 | Sunset Beach, Milnerton | Skin | DOE |
| SAB40 | M | 2004 | Woody Cape | Bone | PEM |
| SAB40 duplicate |  |  |  |  |  |
| SAB42 | M | 1977 | King Beach, PE | Bone | PEM |
| SAB44 | U | 1965 | Gouritz River Mouth | Bone | PEM |
| SAB45 | U | 1952 | Gamtoos River Mouth | Bone | PEM |
| SAB45 duplicate |  |  |  |  |  |
